# A Voxel-Based Radiographic Analysis Reveals the Biological Character of Proneural-Mesenchymal Transition in Glioblastoma

**DOI:** 10.3389/fonc.2021.595259

**Published:** 2021-03-17

**Authors:** Tengfei Qi, Xiangqi Meng, Zhenyu Wang, Xinyu Wang, Nan Sun, Jianguang Ming, Lejia Ren, Chuanlu Jiang, Jinquan Cai

**Affiliations:** ^1^Department of Neurosurgery, The Second Affiliated Hospital of Harbin Medical University, Harbin, China; ^2^Department of Microbiology, Tumor and Cell Biology (MTC), Biomedicum, Karolinska Institutet, Stockholm, Sweden

**Keywords:** voxel-based lesion-symptom mapping, proneural subtype, mesenchymal subtype, magnetic resonance imaging, glioblastoma

## Abstract

**Introduction:** Proneural and mesenchymal subtypes are the most distinct demarcated categories in classification scheme, and there is often a shift from proneural type to mesenchymal subtype in the progression of glioblastoma (GBM). The molecular characters are determined by specific genomic methods, however, the application of radiography in clinical practice remains to be further studied. Here, we studied the topography features of GBM in proneural subtype, and further demonstrated the survival characteristics and proneural-mesenchymal transition (PMT) progression of samples by combining with the imaging variables.

**Methods:** Data were acquired from The Cancer Imaging Archive (TCIA, http://cancerimagingarchive.net). The radiography image, clinical variables and transcriptome subtype from 223 samples were used in this study. Proneural and mesenchymal subtype on GBM topography based on overlay and Voxel-based lesion-symptom mapping (VLSM) analysis were revealed. Besides, we carried out the comparison of survival analysis and PMT progression in and outside the VLSM-determined area.

**Results:** The overlay of total GBM and separated image of proneural and mesenchymal subtype revealed a correlation of the two subtypes. By VLSM analysis, proneural subtype was confirmed to be related to left inferior temporal medulla, and no significant voxel was found for mesenchymal subtype. The subsequent comparison between samples in and outside the VLSM-determined area showed difference in overall survival (OS) time, tumor purity, epithelial-mesenchymal transition (EMT) score and clinical variables.

**Conclusions:** PMT progression was determined by radiography approach. GBM samples in the VLSM-determined area tended to harbor the signature of proneural subtype. This study provides a valuable VLSM-determined area related to the predilection site, prognosis and PMT progression by the association between GBM topography and molecular characters.

## Introduction

Glioblastoma (GBM), mainly diagnosed by magnetic resonance imaging (MRI) and accurate pathological examination, is the most aggressive brain tumor and indicates a poor prognosis (1). The current standard of care refers to maximal surgical resection followed by local radiotherapy and chemotherapy. However, the tumor evolves rapidly in the progression, which is linked to resistance to adjuvant treatment (2, 3).

As a non-invasive checking method, MRI is capable of conducting qualitative and quantitative analysis with specific phenotypic imaging features, to associate with potential prognosis and characteristics (4). The genomic characteristics of heterogeneous MRI features in GBM are investigated and determined by scholars in a growing number of studies, which provide chances for grouping, prognostication and innovation of targeted therapies (5, 6). As a newly developed terminology, Visually Accessible Rembrandt Images (VASARI) feature set (https://wiki.nci.nih.gov/display/CIP/VASARI) incorporates various visible subjective imaging features, which is designed to normalize grading of the distinct features of gliomas on MRI, containing different grades criteria corresponding to diverse score to depict severity (7).

GBM with specific anatomical region shows similarity in genomic alterations and gene expression patterns (8). Voxel-based lesion-symptom mapping (VLSM) approach is one of the most common method to explore the relationship between GBM topography in MRI and lesion-behavior based on voxel-by-voxel method (9). In consideration of tumorigenesis and progression characteristics of GBM, VLSM is widely used to investigate tumor location involved in occupation and other affected secondary diseases (10). VLSM analysis is reported to identify the genomic alterations and the object-action dissociation in studies (11, 12).

Both MRI features and transcriptome analysis reveal distinct subtypes of GBM with different clinical and molecular characteristics (13). The Cancer Genome Atlas (TCGA, https://portal.gdc.cancer.gov/) has described a robust gene expression-based molecular classification of GBM including proneural, mesenchymal, neural, and classical subtypes (14). Notably, there is a consensus that proneural and mesenchymal subtypes are the most distinct demarcated categories among different subtypes (15–17). Proneural subtype is generally regarded as a common precursor of several molecular subtypes, while mesenchymal subtype indicates worst prognosis and lowest tumor purity (18–20). It is worth noting that there is plasticity for proneural subtype, and it has been proved to be with a tendency toward proneural-mesenchymal transition (PMT) progression during glioma progression (21, 22). Halliday found that a marked shift away from a proneural expression pattern toward a mesenchymal one in GBM (23). PMT progression may represent for GBM the equivalent of epithelial–mesenchymal transition (EMT) process associated with other aggressive cancers (24). The EMT process refers to transdifferentiation of epithelial cells into motile mesenchymal cells in tumor progression and metastasis, which is mediated by plenty of key transcription factors (25). To date, GBM topography in proneural or mesenchymal subtypes is not elaborately analyzed in previous studies.

In our study, we screened 223 samples in TCIA (The Cancer Imaging Archive, TCIA, http://cancerimagingarchive.net) database and elucidated the radiogenomic signatures in GBM via common MRI alignment. As for GBM topography for subtypes is not clear to date, we used VLSM method to evaluate the predilection sites of proneural and mesenchymal subtypes. The combination of VLSM method and VASARI features was applied to analyze overall survival (OS) and other significant information based on the VLSM-determined area, contributing to understand the genomics pathogenesis potentially.

## Results

### Demographic Characteristics

**Figure 1:**
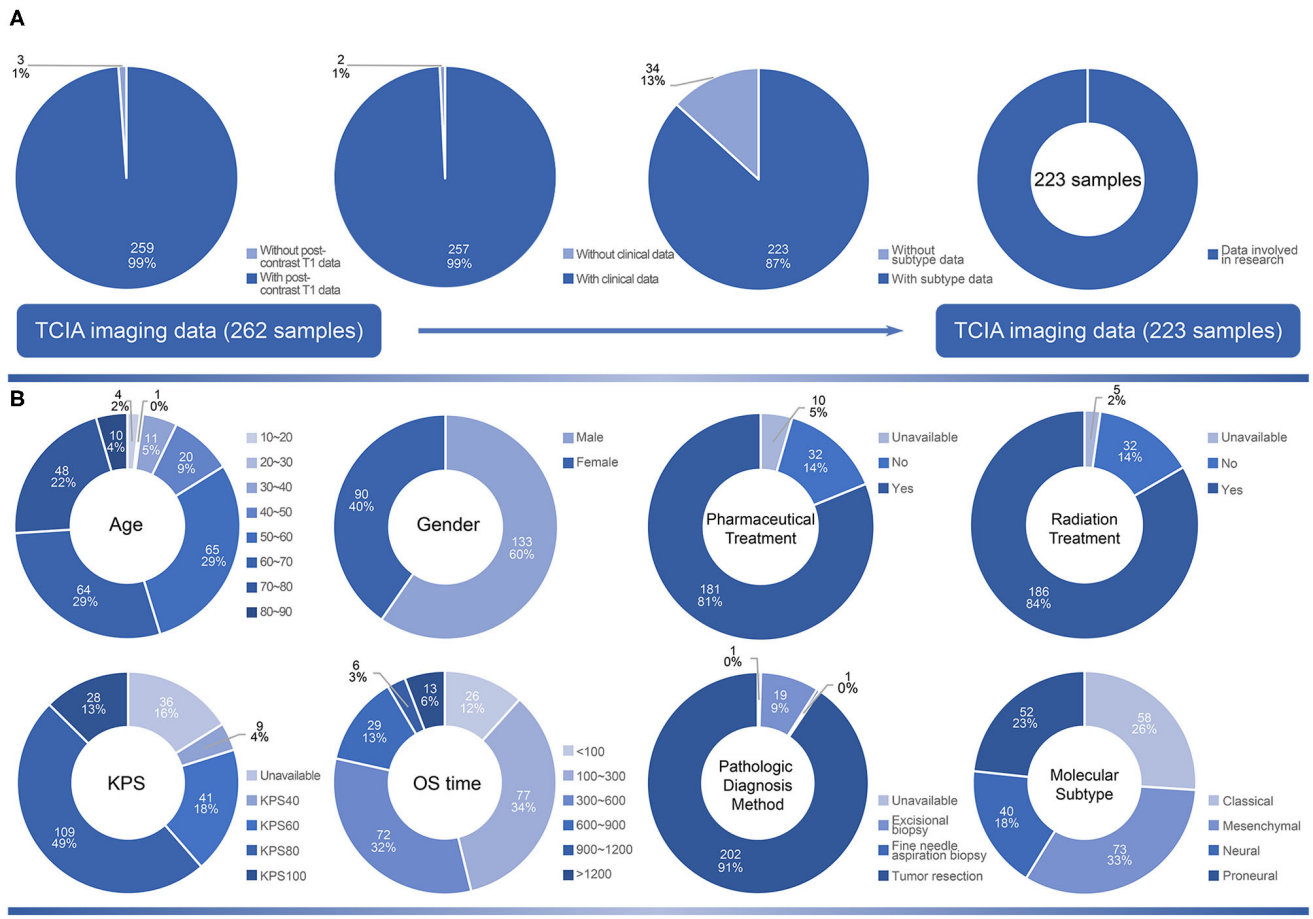
The selection criteria of the study and demographic characteristics of samples. **(A)** The original 262 samples collected from TCIA. According to the availability of post-contrast T1 image, clinical data and subtype data, 223 samples were included in the study. **(B)** The distribution of clinical variables including age, gender, pharmaceutical treatment, radiation treatment, KPS, OS time, pathologic diagnosis method and molecular subtype in 223 samples.

The distribution of 223 samples enrolled into the study were illustrated in Figure 1 (Supplementary Table 1). In the age distribution of patients, there were 65 patients (29% of 223) were between 50 and 60 years old. In the gender distribution of patients, there were 90 females (40% of 223) and 133 males (60% of 223). As high as 81 an d84% of the patients had received pharmaceutical and radiation treatment, respectively, while only 5 and 2% lack relevant information, respectively. Karnofsky performance status (KPS) value of 80 was in 49% of the patients. The OS time was in a range of 4–1,730 days. The survival of 100–300 days (34%) harbored the highest proportion. The patients who had received tumor resection procedure were 91%, and 19% of the patients only experienced excisional biopsy to acquire pathologic information. In the molecular subtypes distribution, mesenchymal subtype had a proportion of 33%, whereas proneural, neural and classical subtypes were 23, 18, and 26% of total, respectively.

GBM topography at lobe level was summarized in Table 1. The predilection site of samples involved with temporal lobe (75.34% of total, 80.77% of proneural subtype, 68.50% of mesenchymal subtype, 82.50% of neural subtype, 74.13% of classical subtype). The proportion of single temporal lobe (21.92%) involved in mesenchymal subtype was a little higher than other subtypes. Samples with mesenchymal subtype had the highest percentage outside temporal lobes (31.51%). There were 103 samples with tumor located in the right hemisphere and 104 samples with tumor located in the left. All the subtypes indicated no obvious inclination in tumor side.

**Table 1:** The distribution of different subtypes based on tumor locations.

	**Total (*n* = 223)**	**Proneural (*n* = 52)**	**Mesenchymal (*n* = 73)**	**Neural (*n* = 40)**	**Classical (*n* = 58)**
Single temporal lobe, *n* (%)	38 (17.04)	10 (19.23)	16 (21.92)	4 (10.00)	8 (13.79)
Multiple lobes including temporal lobe, *n* (%)	130 (58.30)	32 (61.54)	34 (46.58)	29 (72.5)	35 (60.34)
Other locations, *n* (%)	55 (24.66)	10 (19.23)	23 (31.51)	7 (17.5)	15 (25.86)
Left, *n* (%)	103 (46.19)	29 (55.77)	36 (49.32)	14 (35.00)	24 (41.38)
Right, *n* (%)	104 (46.64)	21 (40.38)	33 (45.21)	21 (52.50)	29 (50.00)
Bilateral, *n* (%)	16 (7.17)	2 (3.85)	4 (5.48)	5 (12.50)	5 (8.62)

**Figure 2:**
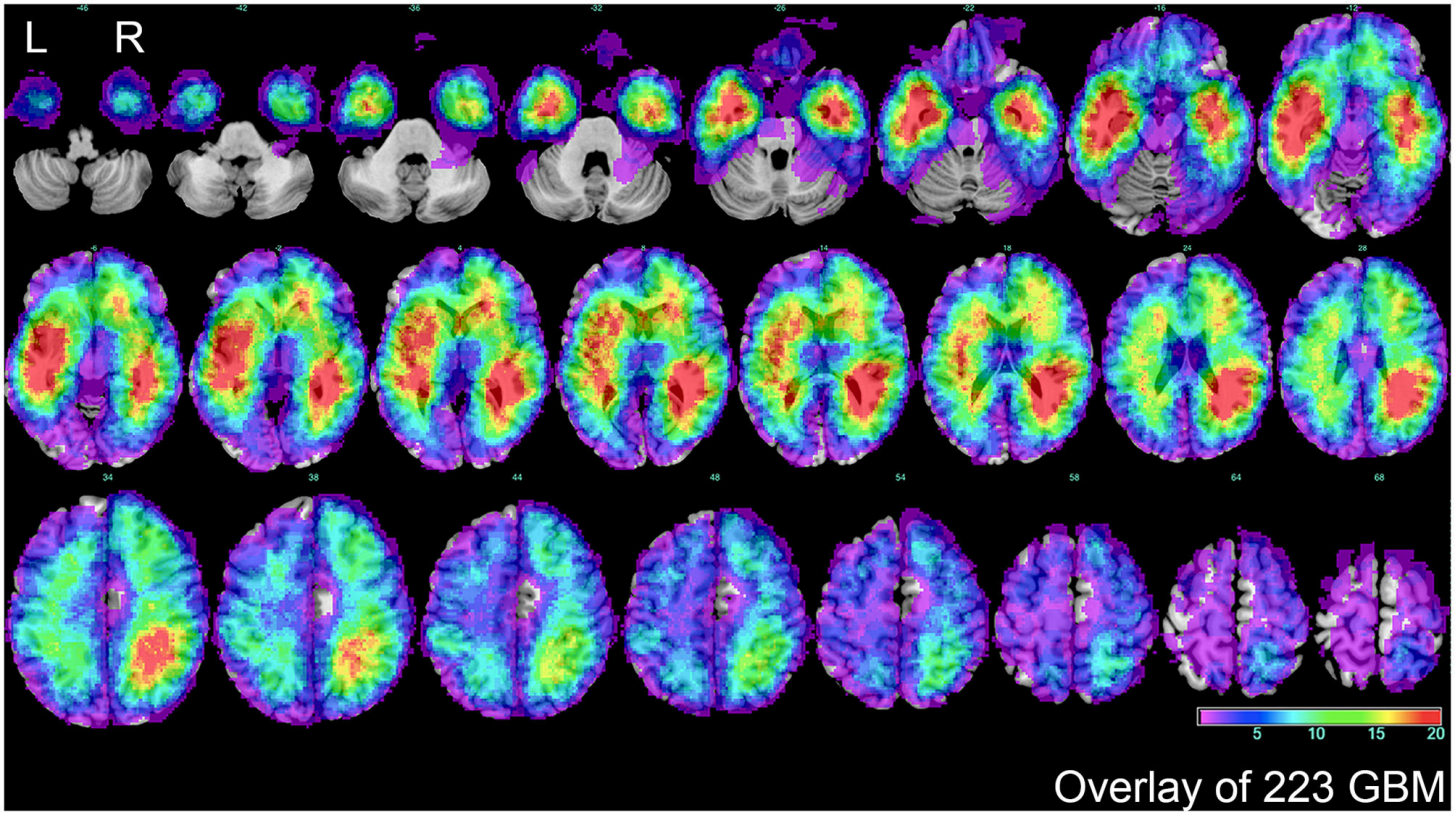
The overlap of total GBM lesions. VOIs of 223 GBM samples included in the study were overlaid on standard template. The color scale indicated the amount of the VOIs overlap from violet (1 case) to red (more than 20 cases). The distribution showed that a majority of GBM located in periventricular or subventricular region. L represented left side, and R represented right side.

To further explore characteristics of GBM topography, the Volume of interests (VOIs) of the whole patient cohort were overlapped on the standard template (Figure 2). GBM topography was evenly distributed in periventricular zone and adjacent to the subventricular zone. In addition, there was no significant discrepancy between tumors in the left and those in the right side.

### VLSM-Determined Area

**Figure 3:**
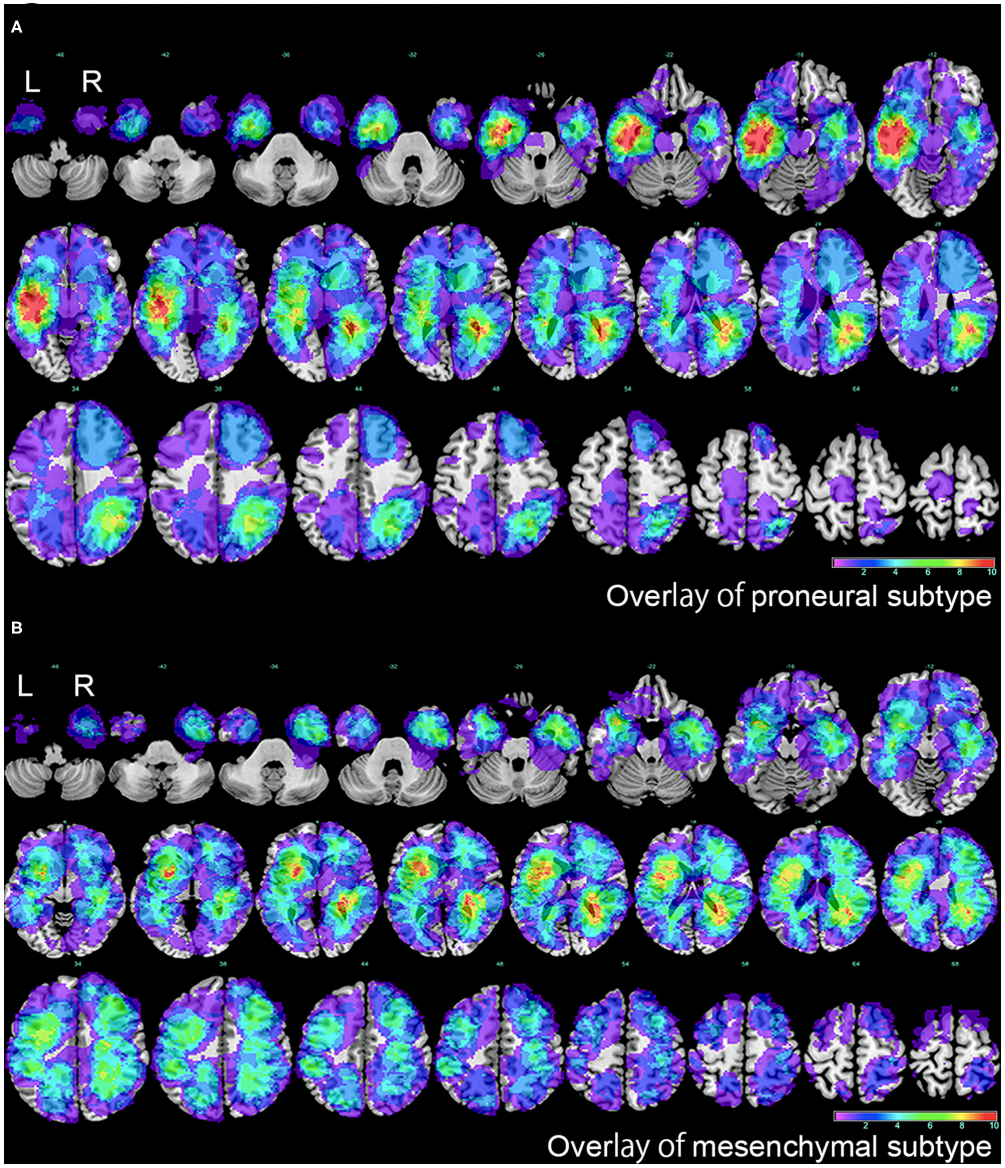
The overlap of proneural and mesenchymal subtypes of GBM. **(A)** The overlap of proneural subtype of GBM. The color scale indicated the amount of the VOIs overlap, from violet (1 case) to red (more than 10 cases). **(B)** The overlap of mesenchymal subtype of GBM. The color scale indicated the amount of the VOIs overlap, from violet (1 case) to red (more than 10 cases). L represented left side, and R represented right side.

Firstly, separated tumor overlays were performed to detect GBM topography of proneural and mesenchymal subtypes, and we finally found that compared with integral overlay displayed in Figure 2, the overlays of two subtypes had diversities (Figures 3A,B). On one hand, overlay on the right side of the two subtypes resembled each other and showed a favorable agreement to integral overlay. On the other hand, two groups indicated different distribution on the left side. High frequency of tumor occurrence of the mesenchymal subtype located forward and close to frontal lobe, while hotspot of the proneural subtype located backwards in cerebral hemisphere and near occipital lobe. These results suggested that the two subtypes had a different tumorigenesis in topography but an intimate relation in progression.

**Figure 4:**
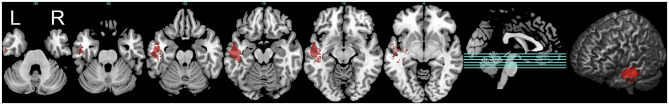
The predilection region of proneural subtype defined by VLSM analysis. The VLSM analysis determined the predilection regions of proneural subtype in the left inferior temporal medulla (marked in red). Three-dimensional render of the VLSM-determined area was also illustrated. L represented left side, and R represented right side. This figure only showed the result below an FDR-adjusted threshold (*P* < 0.05).

To further explore PMT progression, we selected 125 samples comprised of 52 proneural and 73 mesenchymal subtypes to conduct VLSM analysis. The normalized lesion maps in proneural and mesenchymal subtypes were calculated independently. VLSM specifically associated proneural subtype with lesions to a cluster of the left inferior temporal medulla, while no significant voxel was found for mesenchymal subtype (Figure 4). The results of significant clusters of proneural subtype accorded with previous overlay map of the proneural subtype. According to whether the tumors were in or outside the VLSM-determined area of proneural subgroup, the samples were classified into two groups. Of the 125 samples, there were 33 samples in the VLSM-determined area, and 22 samples (67% of 33) were proneural subtype while 11 samples (33% of 33) were mesenchymal subtype. Among 92 samples that were outside the VLSM-determined area, 30 samples (33% of 92) were proneural subtype, whereas 62 samples (67% of 92) were mesenchymal subtype. We also observed that among 52 proneural samples, 22 samples (42% of 52) were in the VLSM-determined area while 30 samples (58% of 52) were outside the VLSM-determined area. Among 73 mesenchymal samples, 11 samples (15% of 73) were in the VLSM-determined area and 62 samples (85% of 73) were outside the VLSM-determined area (Table 2).

**Table 2:** The distribution of proneural and mesenchymal subtypes in and outside the VLSM-determined area.

	**Proneural subtype**	**Mesenchymal subtype**	**Total**
In the VLSM-determined area	22	11	33
Outside the VLSM-determined area	30	62	92
Total	52	73	125

### Survival Analysis

**Figure 5:**
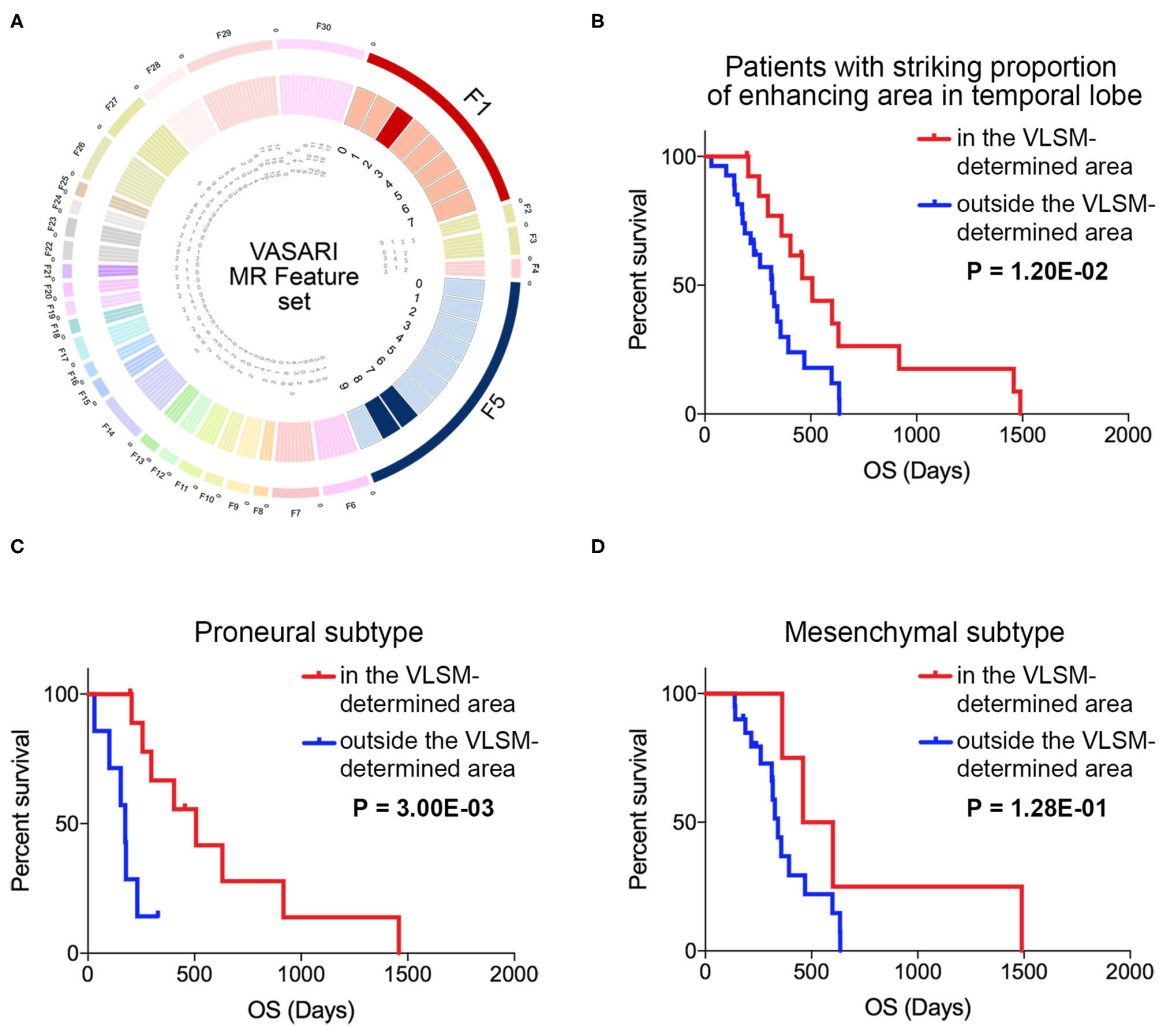
The VASARI feature set used for screening samples and survival analysis in and outside the VLSM-determined area. **(A)** The F1 and F5 in VASARI feature sets were applied. Patients with GBM involved temporal lobe with over 95% proportion of enhancing area were carried out for survival analysis. **(B)** Survival analysis illustrated the significantly different prognosis of patients with GBM in and outside the VLSM-determined area (*P* = 1.20E-02). **(C)** Survival analysis illustrated the significantly different prognosis of patients with proneural subtype of GBM in and outside the VLSM-determined area (*P* = 3.00E-03). **(D)** Survival analysis illustrated the prognosis of patients with mesenchymal subtype of GBM in and outside the VLSM-determined area (*P* = 1.28E-01). *P*-value was determined by log-rank test.

Survival analysis was carried out to find whether the VLSM-determined area could serve as a prognostic role. Initially, to exclude some recognized factors that influence survival outcome obviously, we included samples with standard tumor resection, radiotherapy and pharmaceutical treatment to make the survival result more persuasive. Since contrast enhancement of MRI was associated with survival (26), we selected variable F5 of VASARI feature set to remove the bias of contrast enhancement on survival. In this study, 95% proportion of enhancing area is defined as the cut-off value of striking enhancement. Another variable F1 was also chosen as a reference, and GBM samples involved with temporal lobe were selected (Figure 5A). Kappa consistency test was executed before survival analysis. The results of inter-rater analysis for VASARI features indicated excellent agreement, and kappa values of F1 and F5 were 0.923 and 0.842, respectively (Table 3). Log-rank survival analysis showed that GBM samples in the VLSM-determined area had longer OS time compared with those outside the VLSM-determined area (*P* = 1.20E-2, Figure 5B). Then the data based on proneural or mesenchymal subtype were subdivided to find the significant difference of survival outcome. Proneural subtype in the VLSM-determined area predicted a longer OS time (*P* = 3.00E-03, Figure 5C), while in mesenchymal subtype, there was no significant difference between GBM samples in and outside the VLSM-determined area in survival analysis (*P* = 1.28E-01, Figure 5D).

**Table 3:** Inter-observer analysis for VASARI imaging features used in the study.

**VASARI imaging features**	**Description**	**Options**	**Kappa value**
Tumor location (F1)	Location of lesion geographic epicenter	0 = – 1 = Frontal 2 = Temporal 3 = Insular 4 = Parietal 5 = Occipital 6 = Brainstem 7 = Cerebellum	0.923
Proportion enhancing (F5)	Enhancing proportion of the entire tumor	0 = – 1 = n/a 2 = None (0%) 3 = <5% 4 = 6–33% 5 = 34–67% 6 = 68–95% 7 = > 95% 8 = All (100%) 9 = Indeterminate	0.842

### Clinical Variables, EMT Process, and Tumor Purity

**Figure 6:**
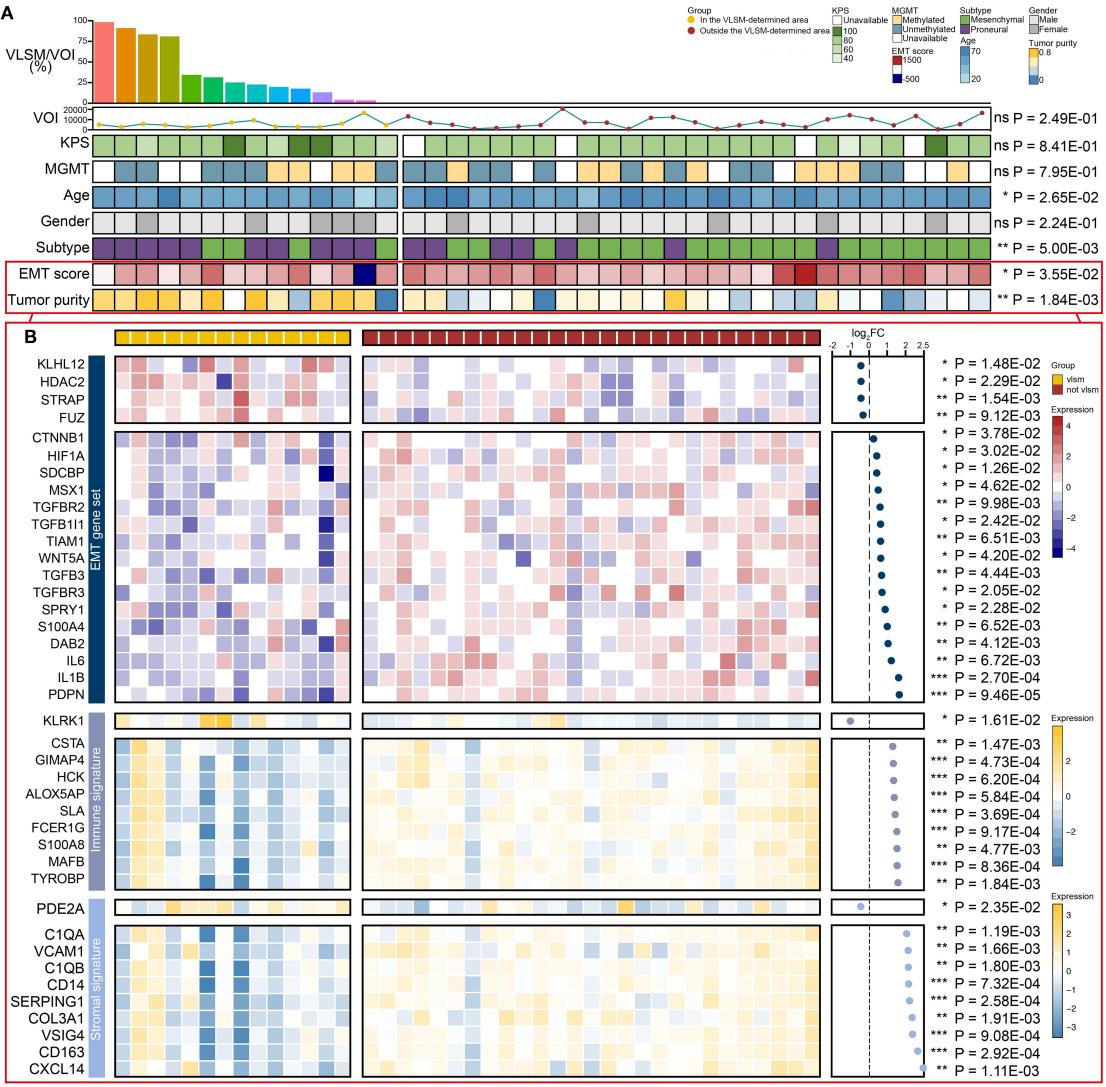
The comparison of EMT score, tumor purity, clinical variables and differential expressed genes between GBM in and outside the VLSM-determined area. **(A)** The EMT score, tumor purity and the clinical variables including VLSM/VOI, VOI, KPS, MGMT, age, gender, subtype were used. The histogram presented VLSM/VOI in descending order. The line chart presented the VOIs. The heatmaps presented differences between GBM in and outside the VLSM-determined area. **(B)** The heatmaps showed the differential expressed genes of EMT gene set, immune signature and stromal signature of GBM in and outside the VLSM-determined area. The scatter plot presented the fold change of differential expressed genes in logarithmic form. *P*-value was determined by Student's *t*-test. Significant results were presented as ns non-significant, *P < 0.05, **P < 0.01, or ***P < 0.001.

Apart from clinical variables, the expression profiling data in TCGA was used to calculate the corresponding signal pathway score by single-sample Gene Set Enrichment Analysis (ssGSEA) to evaluate EMT process and tumor purity of samples (Figure 6). For clinicopathological characteristics, age, subtype, EMT score and tumor purity were of significant association in and outside the region (Figure 6A). VOI, KPS, MGMT and gender showed no significant difference (Supplementary Table 2). VLSM/VOI was illustrated as histogram in descending order. Age distribution showed that patients with GBM outside the VLSM-determined area were older than those in the VLSM-determined area. The proportion of proneural subtype was higher in the VLSM-determined area, while samples in mesenchymal subtype had preference locating outside the VLSM-determined area. Compared with samples in the VLSM-determined area, samples outside the VLSM-determined area had a higher EMT score, which indicated a tendency to mesenchymal phenotype. As for tumor purity, we calculated immune signature and stromal signature together. The top 10 genes with significant differential expression were presented in the image (Figure 6B, Supplementary Table 3). In EMT gene set, KLHL12, HDAC2, STRAP, and FUZ were over-expressed in VLSM-determined area, and other genes such as CTNNB1, HIF1A, and IL6 were over-expressed outside the VLSM-determined area. These results demonstrated that there were significant differences of EMT process and tumor purity between GBM in and outside the VLSM-determined area, indicating the potential PMT progression. We also analyzed the immune cells infiltration between GBM in and outside the VLSM-determined area to further discuss the changes of microenvironment in PMT progression. The infiltrations of T cells CD8 and T cells follicular helper were significantly down-regulated in GBM outside the VLSM-determined area compared with those in GBM in the VLSM-determined area (T cells CD8, *P* = 1.00E-02; T cells follicular helper, *P* = 1.00E-02; Student's *t*-test, Supplementary Table 4).

## Discussion

GBM is categorized into four subtypes (proneural subtype, mesenchymal subtype, classical subtype and neural subtype) based on molecular and phenotypic differences. For instance, mesenchymal subtype has higher rates of proliferation *in vitro* and is markedly resistant to radiotherapy compared with proneural subtype (27). PMT progression widely exists in the process of GBM progression due to the invasive mechanism and activation potential of mesenchymal feature of proneural subtype (19). In this study, we found potential relevance between proneural subtype and mesenchymal subtype on GBM topography based on overlay and VLSM analysis, and survival analysis revealed that patients with GBM in the VLSM-determined area survived longer than patients with GBM outside the VLSM-determined area. Furthermore, the results of EMT score and tumor purity suggested a potential PMT progression between GBM in and outside the VLSM-determined area further. Samples with GBM in and outside the VLSM-determined area harbored the signature of proneural subtype and mesenchymal subtype in PMT progression, respectively. In previous study, the PMT progression is determined by specific genomic methods generally, while this study discussed the subject in radiography approach and presented ideas related to different subtypes (28, 29).

The non-invasive method of MRI materials provides effective mean to explore different molecular genetic signatures (4). Tumor location can be associated with the genetic profile of tumor precursor cells (14). In our study, 223 samples with definite subtypes were selected to conduct tumor overlay maps. The original GBM overlay result indicated the overlays with higher proportion were around periventricular or subventricular zone, which suggested the result was associated with the origin of brain tumors (30). Proneural subtype and mesenchymal subtype are the most distinct subtypes, which suggests a biological significance in tumor biology and overall survival (31–33). According to overlay of the VOIs in proneural and mesenchymal subtypes gathered with diverse predilection site, we explored the anatomical characteristics of the two subtypes. The predilection area of proneural glioma was involved with left middle and left inferior temporal gyrus. The brain temporal lobe plays a role in recognition of specific objects and processing with visual stimuli (34), and is one of the most predilection sites for GBM in adults (35). Proneural subtype has classical events in the robust classification scheme including TP53 mutation, and can be a driver of initial oncogenic events and influenced by a variety of genomic factors in tumor initialization and progression (14, 18). The predilection area of mesenchymal glioma located forward and close to brain frontal lobe. Gliomas located in the frontal lobe have symptoms including dementia, personality change, gait disturbance, expressive aphasia and others (36). The mesenchymal gliomas expressed properties such as reduced cell polarity, increased pseudopodia formation, cell motility and invasion, upregulation of EMT markers (19). The overlays of the VOIs in proneural and mesenchymal subtypes gathered with diverse predilection site. Therefore, we employed VLSM analysis to explore the anatomical characteristics of the two subtypes. The overlays of the VOIs in proneural subtype and mesenchymal subtype gathered with diverse predilection site. Therefore, we employed VLSM analysis to explore the anatomical characteristics of the two subtypes.

VLSM is a different method from overlay, which can provide statistical significance for the observed difference based on voxel level and bring forward to evaluate the relationship between lesions and clinical symptoms (37). In present study, proneural subtype was confirmed to be related to left inferior temporal medulla. We morphologically defined a VLSM-determined area as inclination tumorigenesis of proneural subtype. However, no significant voxel was detected in mesenchymal subtype. The overlay images provided evidence that mesenchymal subtype GBM had a relative diffused distribution. This characteristic might be explained by the absence of diffused growing patterns of mesenchymal subtype (38).

In the analysis of MRI radiography, tumor contrast enhancement is applied to predict GBM prognosis and malignancy, which can be easily distinguished, resulting to the narrowing of subjective error of visual inspection and facilitating feasibility or availability (39, 40). In this study, we employed VASARI feature set to formulate proportion of enhancing area for ensuring the normalized visual feature. GBM involved temporal lobe with over 95% proportion of enhancing area, as well as other potential factors involved in patient's prognosis such as tumor resection, radiation treatment and pharmaceutical adjuvant, were used for sample filtration to further analysis of VLSM-determined area. GBM samples in the VLSM-determined area had longer OS time compared with those outside the VLSM-determined area. For proneural subtype, samples in the VLSM-determined area had longer survival time than those outside the VLSM-determined area. However, there was no significant difference for mesenchymal subtype, which could be explained by the limited amount of data.

Different metastasis and immune related genes manifest differential expression patterns among GBM subtypes (41, 42). Mesenchymal subtype is characterized by an increased immune cell presence compared to proneural subtype (43). The existence of PMT progression can influence survival time, the sensitivity to radiotherapy and chemotherapy, potential target in gene therapy and tumor immunity (44). Quiescent GBM cells gain malignant potency by engaging a mesenchymal shift that resembles EMT process and increases invasive behaviors (45). Tumor purity refers to the proportion of cancer cells in a tumor sample and is negatively correlated with EMT process and immune activity (46). Cases with low tumor purity are more likely to be related to malignant entities and have reduced survival time, which resembles PMT progression, indicating a worsening process and enabling the tumor incline to obtain characters of mesenchymal subtype (24). Besides, PMT progression indicates a worsening process and enables the tumor incline to obtain characters of mesenchymal subtype (24, 47). Compared with proneural subtypes, mesenchymal subtype had the lower purity score, indicating a lower tumor purity with the infiltration of non-neoplastic cells into this subtype (19, 48). In order to assess the PMT progression of GBM, we compared EMT process and tumor purity by expression profiling data of samples in and outside the VLSM-determined area via ssGSEA. Samples outside the VLSM-determined area had higher EMT scores than those in the VLSM-determined area, representing that the EMT process were upregulated in GBM outside the VLSM-determined area. Low tumor purity, indicating poor prognosis and an intense immune phenotype (49), was detected in samples outside the VLSM-determined area. The diverse pattern of the presence of stromal and immune cells across tumor types more broadly illustrates the impact of the tumor microenvironment on tumorigenesis and homeostasis (46). In our study, the samples outside the VLSM-determined area had lower tumor purity, higher stromal and immune signature gene expression than samples in the VLSM-determined area, indicating a higher infiltration of stromal and immune cells in glioma tissues (50). Immune signature genes, such as S100A8, had been shown to be pro-tumorigenic by inducing infiltration of myeloid derived suppressor cells (MDSCs) (51) or suppressing T cell function at the tumor site (52). High expression of stromal signature genes, such as COL3A1, is correlated with poor prognosis in glioblastoma (53). These results demonstrated that the different microenvironment could regulate the malignant progression between GBM in and outside the VLSM-determined. In addition, the microenvironment plays a key role in PMT progression. GBM subtypes shift from one to another one upon changes in the microenvironment. The average percentage of the different ratio in 22 types immune cells between GBM in and outside the VLSM-determined area were calculated and displayed. According to the result, GBM outside the VLSM-determined area had lower infiltrations of T cells CD8 and T cells follicular helper compared with GBM in the VLSM-determined area. The immune response of patients with glioma are characterized by defects in poor tumor antigen-specific CD8+ T cell responses, and elevated programmed death 1 (PD-1) in CD8+ T cells contributing to the poor prognosis of these patients (54, 55). Low tumor-infiltrating CD8+ T cells were associate with poor progression-free survival (56). T follicular helper cell could activate B cells to facilitate the anti-tumor response (57). These results demonstrated that the different immune cells infiltration between GBM in and outside the VLSM-determined area could regulate the malignant progression of glioma.

In present study, the difference of gene expression patterns between GBM in and outside the VLSM-determined area provided reference to molecular targeting treatment according to different topography (58). Among 141 genes of EMT gene set, 16 genes including CTNNB1, HIF1A, and IL6 were upregulated. CTNNB1, the downstream effector of the canonical WNT signaling pathway, is a key feature of EMT process, which has been identified as a therapeutic target for GBM (59). Multiple HIF1A-responsive EMT regulators in cancers is sufficient to induce all stages of cancer spread, including invasion, intravasation, and distant extravasation (60). Cytokines such as IL6 are capable of inducing EMT process by downregulation of E-cadherin and upregulation of Vimentin (61–63). The top 10 genes of differential expressed genes in stromal signature and immune signature were also of vital significance in PMT progression of GBM. Single cell analysis of immune cells in GBM showed that S100A8/9 (macrophages markers) was highly expressed in immune cells in the tumor core, indicating that the infiltration of immune cells within the mesenchymal subtype (19). CXCL14 enhances the sphere-forming ability of GBM cells, overexpresses in mesenchymal tumors and is responsible for tumor onset, growth and recurrence (64).

We realized the limitations of our techniques. Firstly, the VLSM method regarded each voxel as being independent and separated from other adjacent voxels, which may influence the calculation of involved regions. In addition, although the general MR imaging sequences ensure the reliability or reproducibility of the study, senior scans (such as DWI, DTI, MRS) are beneficial for further study of intratumoural transcriptional heterogeneity with novel algorithm.

Our study demonstrated a valuable VLSM-determined area related to the predilection site, prognosis and PMT progression by radiography approach. GBM involved in VLSM-determined area exhibited the characters of proneural subtype. The results also revealed the differences of EMT process and tumor purity among GBM in and outside the VLSM-determined area.

## Materials and Methods

### Data Collection

Original data used in this research were provided by TCGA, an open resource containing comprehensive genomics information on various cancers. TCGA data collection founded by the cooperation between National Cancer Institute (NCI) and several institutions, publicly available in TCIA database, was selected to explore the connection between GBM phenotypes and radiographs, for data were matched to store in TCGA and TCIA (65). All information was available in an open manner, and no institutional review board or Act approval was essential. Imaging data comprised various general sequences such as T1-weighted, T2-weighted images, and other advanced MRI scans. Among different original pre-operative multimodal MRI scans, post-contrast T1-weighted and other available sequences such as T2-weighted images were employed in present study. Clinical data and molecular genetic data included gender, OS time, age, KPS, and other information (66).

MRI data of total 262 GBM samples including multiple sequences were acquired initially. The exclusion criteria referred to sample lacks post-contrast T1-weighted, subtype classification, and clinical features. After summarizing the entire data together, we identified 223 samples who had relevant variables available finally. A flowchart of the number of samples included or excluded for each analysis was shown in Supplementary Figure 1.

### Imaging Processing

Prior to imaging processing, the original imaging dataset was evaluated by VASARI feature set, which served as a semi-quantitative imaging analysis for describing visual features on MRI (67). In this study, two variables describing topography of brain lobes of lesion and proportion of enhancing area were used, which were visually estimated by observers and divided into separate categories. For each sample, the images were evaluated by a neurosurgeon and a neuroradiologist independently and they knew nothing about other data. The third experienced neurosurgeon made the final decision judged by multiple MRI sequences when there was a discrepancy. All the observers learned the visual examples of scoring consensus in advance to ensure agreement. Kappa consistency test was used to check inter-observer variation.

After radiography materials were downloaded, format transformation was carried out to acquire NIfTI format profiles. The neurosurgeon and neuroradiologist manually draw the lesion map on post-contrast T1-weighted image in each axial slice by the usage of MRIcron (http://www.sph.sc.edu/comd/rorden/mricron) to delineate the boundary of the tumor. If there was an evident discrepancy (the ratio of VOI between two observers > 0.05), the VOIs of the samples would be rechecked by another neurosurgeon to make the final decision. Then VOIs of all samples were collected. The sequences were normalized into the stereotactic Montreal Neurological Institute (MNI) standard space. With Statistical Parametric Mapping 8 (SPM 8, http://www.fil.ion.ucl.ac.uk/spm/software/spm8) implemented in MATrix LABoratory (MATLAB, Mathworks, Natick, MA), images and generated VOIs were registered to brain atlas and normalized at the MNI space using the standard normalization algorithm followed by examination of visual inspection (68). To explore the distribution of GBM and correlations among different subtypes, all normalized VOIs were summed up together to get the overlay maps on ch2bet template.

### VLSM Method

VLSM method was applied to relate lesion map to manifestation of proneural and mesenchymal subtype in MRIcron software by Non-parametric Mapping Statistics (NPM, http://www.mccauslandcenter.sc.edu/mricro/mricron/) (69). Subsequently, a spreadsheet was generated to link the lesion to subtype of each sample. Non-parametric Liebermeister test was used to perform statistical comparisons on voxel-wise base, which were performed at each voxel using specific subtypes as dependent variable (binary measure). *Z*-value corresponding to significant *P* level indicated the minimum threshold of significant topography, and a higher statistical output meant stronger association between predilection site of GBM and specific subtypes. Analysis was only computed on voxels damaged > 5%. NPM false discovery rate (FDR) correction for multiple comparisons was applied. VLSM maps were displayed on ch2bet template with a statistical significance (*P* < 0.05).

### Estimation of EMT Process and Tumor Purity

Expression profiling data from TCGA was used to evaluate EMT enrichment and tumor purity (70). SsGSEA was applied to get the enrichment scores of EMT gene set and tumor purity associated gene sets for each sample (71). A single sample's genes expression profiling data from the space of single genes were projected onto the space of every gene set by ssGSEA, and each enrichment score was on behalf of the degree to which the genes in every gene set were coordinately up-regulated or down-regulated within a sample (72).

### Statistics

Statistical analysis (VLSM analysis excluded) was carried out by IBM SPSS statistics and GraphPad Prism. All results were shown as mean ± standard deviation or number of observations and percentages. Kappa consistency test was applied to evaluate consistency in the diagnosis of VASARI scores between different observers (73), and the kappa values > 0.8, in the range of 0.6–0.8, and <0.6 indicated excellent, good and poor agreement, respectively. χ^2^-test was used to detect the distribution of several attributes on categorical variables, while Student's *t*-test checked the differences between two groups on continuous variables. *R* packages, such as pheatmap, limma and affy, were used to produce figures and calculate differential expressed genes. Regarding to survival analysis, Kaplan-Meier curve and log-rank test were applied to describe OS time. *P* < 0.05 was considered statistically significant.

## Data Availability Statement

Publicly available datasets were analyzed in this study. This data can be found at: http://cancerimagingarchive.net, TCIA.

## Ethics Statement

Ethical review and approval was not required for the study on human participants in accordance with the local legislation and institutional requirements. Written informed consent for participation was not required for this study in accordance with the national legislation and the institutional requirements.

## Author Contributions

CJ and JC designed the experiments. TQ and XM performed the experiments. TQ, XM, and JC analyzed the data and wrote the manuscript. ZW, XW, NS, JM, and LR contributed to materials and analysis tools. All authors revised the manuscript.

## Conflict of Interest

The authors declare that the research was conducted in the absence of any commercial or financial relationships that could be construed as a potential conflict of interest.
