# Supplementary material for: A Voxel-Based Radiographic Analysis Reveals the Biological Character of Proneural-Mesenchymal Transition in Glioblastoma
Source: Front Oncol. 2021 Mar 17;11:595259. doi: 10.3389/fonc.2021.595259 (PMC8010193; doi:10.3389/fonc.2021.595259)
Supplement: Supplementary file 1 [file Data_Sheet_1.pdf]

# *Supplementary Material*

## Supplementary Tables

**Supplementary Table 1. Clinical characteristics of 223 patients.**

| Characteristics | | Value (amount, % /range) |
|---|---|---|
| Age (years) | | 60.8 ± 13.3 |
| Gender (Female/Male) | | |
| | Female | 90, 40% |
| | Male | 133, 60% |
| KPS (score) | | |
| | 40 | 36, 16% |
| | 60 | 28, 13% |
| | 80 | 109, 49% |
| | 100 | 41, 18% |
| | Unavailable | 9, 4% |
| OS time (days) | | |
| | <100 | 26, 12% |
| | 100-300 | 77, 34% |
| | 301-600 | 72, 32% |
| | 601-900 | 29, 13% |

| | | |
|---|---|---|
| | 901-1200 | 6, 3% |
| | >1200 | 13, 6% |
| Pharmaceutical treatment (Yes/No) | | |
| | Yes | 181, 81% |
| | No | 32, 14% |
| | Unavailable | 10, 5% |
| Radiation treatment (Yes/No) | | |
| | Yes | 186, 84% |
| | No | 32, 14% |
| | Unavailable | 5, 2% |
| Pathologic diagnosis method | | |
| | Tumor resection | 202, 91% |
| | Excisional biopsy | 19, 9% |
| | Fine needle aspiration biopsy | 1, 0% |
| | Unavailable | 1, 0% |
| Molecular subtype | | |
| | Proneural | 52, 23% |
| | Mesenchymal | 73, 33% |
| | Neural | 40, 18% |

| | | |
|---|---|---|
| | Classical | 58, 26% |
| IDH1 status | | |
| | Wild-type | 178, 80% |
| | Mutant | 1, 0% |
| | Unavailable | 44, 20% |
| MGMT promoter status | | |
| | Methylated | 69, 31% |
| | Unmethylated | 72, 32% |
| | Unavailable | 82, 37% |
| Tissue source institution | | |
| | MD Anderson Cancer Center | 24, 11% |
| | Henry Ford Hospital | 60, 27% |
| | UCSF | 30, 13% |
| | Duke | 22, 10% |
| | Emory University | 25, 11% |
| | Case Western | 33, 15% |
| | Fondazione IRCCS Instituto Neuroligico C. Besta | 15, 7% |
| | Thomas Jefferson University | 14, 6% |

**Supplementary Table 2. The comparison of clinical variables, EMT score and tumor purity between GBM in and outside the VLSM-determined area.**

| | Sample | VLSM/VOI | VOI | KPS | MGMT | Age | Gender | Subtype | EMT score | Tumor purity |
|---|---|---|---|---|---|---|---|---|---|---|
| VLSM-determined area involved | TCGA-08-0353 | 97.99 | 5287 | 80 | Unavailable | 58.4 | Male | Proneural | 547.87 | 0.83 |
| | TCGA-76-6191 | 90.62 | 2966 | 80 | Unmethylated | 57.3 | Male | Proneural | 928.53 | 0.78 |
| | TCGA-14-1454 | 82.97 | 5913 | 60 | Unmethylated | 54.5 | Female | Proneural | 961.80 | 0.97 |
| | TCGA-06-0648 | 80.52 | 4815 | 80 | Unavailable | 78 | Male | Proneural | 604.07 | 0.92 |
| | TCGA-06-0238 | 33.88 | 2684 | 80 | Unavailable | 46.6 | Male | Proneural | 852.93 | 0.66 |
| | TCGA-02-0116 | 30.96 | 4027 | 80 | Unmethylated | 51.1 | Male | Mesenchymal | 1164.61 | 1.00 |
| | TCGA-14-0736 | 24.71 | 7089 | 100 | Unmethylated | 49.9 | Male | Mesenchymal | 723.67 | 0.50 |
| | TCGA-19-5956 | 22.21 | 9492 | 80 | Unmethylated | 53.6 | Female | Proneural | 774.85 | 1.00 |
| | TCGA-12-1602 | 19.20 | 3260 | 60 | Methylated | 58.8 | Male | Proneural | 910.07 | 0.67 |
| | TCGA-02-0064 | 17.19 | 3086 | 100 | Methylated | 50 | Male | Mesenchymal | 1124.32 | 0.27 |

| | | | | | | | | | | |
|---|---|---|---|---|---|---|---|---|---|---|
| | TCGA-06-0241 | 12.77 | 2939 | 100 | Unavailable | 66 | Female | Proneural | 684.86 | 0.87 |
| | TCGA-76-4932 | 3.70 | 6335 | 80 | Methylated | 50.5 | Female | Proneural | 945.43 | 0.94 |
| | TCGA-02-0011 | 2.89 | 16661 | 80 | Methylated | 19 | Female | Proneural | -518.19 | 0.81 |
| | TCGA-06-0139 | 0.29 | 4870 | 60 | Unmethylated | 40.3 | Male | Mesenchymal | 987.26 | 0.00 |
| VLSM-determined area uninvolved | TCGA-14-1794 | 0.00 | 13426 | Unavailable | Unmethylated | 59.2 | Male | Proneural | 1101.82 | 0.65 |
| | TCGA-76-6192 | 0.00 | 6910 | 80 | Unmethylated | 74.8 | Male | Proneural | 937.19 | 0.66 |
| | TCGA-06-5412 | 0.00 | 5204 | 80 | Methylated | 78.8 | Female | Mesenchymal | 872.40 | 0.31 |
| | TCGA-19-1389 | 0.00 | 1206 | 80 | Unmethylated | 51.1 | Male | Mesenchymal | 961.21 | 0.44 |
| | TCGA-27-1830 | 0.00 | 2055 | 80 | Unmethylated | 57.9 | Male | Proneural | 1066.16 | 0.53 |
| | TCGA-06-0646 | 0.00 | 3363 | 80 | Unavailable | 61 | Male | Proneural | 907.78 | 0.60 |
| | TCGA-06-0881 | 0.00 | 4786 | 80 | Unmethylated | 50 | Male | Mesenchymal | 1163.65 | 0.01 |
| | TCGA-06-0156 | 0.00 | 20771 | Unavailable | Unavailable | 57.3 | Male | Proneural | 897.11 | 0.59 |

| | | | | | | | | | |
|---|---|---|---|---|---|---|---|---|---|
| TCGA-14-0783 | 0.00 | 7434 | 80 | Methylated | 36 | Female | Mesenchymal | 748.55 | 0.59 |
| TCGA-14-1452 | 0.00 | 7122 | 80 | Methylated | 60.5 | Male | Mesenchymal | 789.79 | 0.54 |
| TCGA-06-0878 | 0.00 | 936 | 80 | Unmethylated | 74.8 | Male | Mesenchymal | 918.58 | 0.61 |
| TCGA-19-2623 | 0.00 | 11772 | 80 | Methylated | 65.3 | Male | Mesenchymal | 724.87 | 0.52 |
| TCGA-14-1825 | 0.00 | 12496 | 80 | Unmethylated | 70.3 | Male | Proneural | 808.43 | 0.93 |
| TCGA-06-1802 | 0.00 | 7461 | 80 | Methylated | 61.7 | Male | Mesenchymal | 895.02 | 0.59 |
| TCGA-06-0149 | 0.00 | 1068 | 80 | Unavailable | 74.8 | Female | Mesenchymal | 868.22 | 0.51 |
| TCGA-06-0141 | 0.00 | 4625 | 80 | Unmethylated | 62.8 | Male | Mesenchymal | 797.31 | 0.31 |
| TCGA-27-2519 | 0.00 | 7926 | 80 | Unmethylated | 48.5 | Male | Mesenchymal | 625.55 | 0.52 |
| TCGA-06-0190 | 0.00 | 5218 | 80 | Unavailable | 62.5 | Male | Mesenchymal | 1278.08 | 0.34 |
| TCGA-19-1385 | 0.00 | 2766 | Unavailable | Methylated | 69.7 | Male | Mesenchymal | 1521.07 | 0.27 |
| TCGA-76-4935 | 0.00 | 10289 | 80 | Methylated | 52.6 | Female | Proneural | 1171.23 | 0.68 |

| | | | | | | | | | |
|---|---|---|---|---|---|---|---|---|---|
| TCGA-14-0789 | 0.00 | 14488 | 40 | Methylated | 54.9 | Male | Mesenchymal | 1037.83 | 0.47 |
| TCGA-06-0143 | 0.00 | 10525 | 60 | Unmethylated | 58.5 | Male | Mesenchymal | 977.28 | 0.51 |
| TCGA-06-0130 | 0.00 | 4636 | 80 | Unmethylated | 54.2 | Male | Mesenchymal | 1092.80 | 0.07 |
| TCGA-06-0189 | 0.00 | 13740 | Unavailable | Unavailable | 55.6 | Male | Mesenchymal | 1192.31 | 0.26 |
| TCGA-06-0168 | 0.00 | 704 | 100 | Unavailable | 59.6 | Female | Mesenchymal | 808.28 | 0.45 |
| TCGA-02-0075 | 0.00 | 5621 | 80 | Methylated | 63.5 | Male | Mesenchymal | 875.22 | 0.49 |
| TCGA-08-0522 | 0.00 | 16621 | 80 | Unavailable | 61.4 | Male | Mesenchymal | 1107.94 | 0.41 |

**Supplementary Table 3. The differential expressed genes of EMT gene set and tumor purity associated gene signatures between samples in and outside the VLSM-determined area.**

| | Gene Symbol | Average Expression (in the VLSM-determined area) | Average Expression (outside the VLSM-determined area) | $log_2FC$ | P value |
|---|---|---|---|---|---|
| EMT gene set | KLHL12 | 5.77 | 5.32 | -0.45 | 1.48E-02 |
| | HDAC2 | 9.10 | 8.65 | -0.45 | 2.29E-02 |
| | STRAP | 11.12 | 10.67 | -0.44 | 1.54E-03 |
| | FUZ | 5.54 | 5.21 | -0.33 | 9.12E-03 |
| | CTNNB1 | 10.00 | 10.24 | 0.24 | 3.78E-02 |
| | HIF1A | 11.40 | 11.80 | 0.41 | 3.02E-02 |
| | SDCBP | 11.35 | 11.79 | 0.44 | 1.26E-02 |
| | MSX1 | 7.07 | 7.59 | 0.52 | 4.62E-02 |
| | TGFBR2 | 5.92 | 6.55 | 0.62 | 9.98E-03 |
| | TGFB1I1 | 7.55 | 8.20 | 0.65 | 2.42E-02 |
| | TIAM1 | 6.17 | 6.82 | 0.65 | 6.51E-03 |
| | WNT5A | 6.99 | 7.65 | 0.66 | 4.20E-02 |
| | TGFB3 | 5.71 | 6.42 | 0.70 | 4.44E-03 |
| | TGFBR3 | 6.20 | 6.93 | 0.73 | 2.05E-02 |
| | SPRY1 | 7.14 | 8.04 | 0.90 | 2.28E-02 |
| | S100A4 | 7.79 | 8.80 | 1.02 | 6.52E-03 |

| | | | | | |
|---|---|---|---|---|---|
| | DAB2 | 7.26 | 8.32 | 1.06 | 4.12E-03 |
| | IL6 | 5.32 | 6.56 | 1.24 | 6.72E-03 |
| | IL1B | 5.77 | 7.41 | 1.64 | 2.70E-04 |
| | PDPN | 7.40 | 9.08 | 1.68 | 9.46E-05 |
| Immune signature | KLRK1 | 5.30 | 4.57 | -0.21 | 1.61E-02 |
| | CSTA | 6.45 | 7.77 | 0.27 | 1.47E-03 |
| | GIMAP4 | 6.87 | 8.21 | 0.26 | 4.73E-04 |
| | HCK | 6.05 | 7.41 | 0.29 | 6.20E-04 |
| | ALOX5AP | 9.24 | 10.63 | 0.20 | 5.84E-04 |
| | SLA | 6.82 | 8.26 | 0.28 | 3.69E-04 |
| | FCER1G | 8.62 | 10.16 | 0.24 | 9.17E-04 |
| | S100A8 | 6.49 | 8.03 | 0.31 | 4.77E-03 |
| | MAFB | 8.97 | 10.52 | 0.23 | 8.36E-04 |
| | TYROBP | 8.80 | 10.40 | 0.24 | 1.84E-03 |
| Stromal signature | PDE2A | 6.40 | 5.91 | -0.11 | 2.35E-02 |
| | C1QA | 8.68 | 10.39 | 0.26 | 1.19E-03 |
| | VCAM1 | 7.20 | 8.97 | 0.32 | 1.66E-03 |
| | C1QB | 9.49 | 11.28 | 0.25 | 1.80E-03 |
| | CD14 | 9.01 | 10.81 | 0.26 | 7.32E-04 |

| | | | | | |
|---|---|---|---|---|---|
| | SERPING1 | 7.38 | 9.23 | 0.32 | 2.58E-04 |
| | COL3A1 | 8.34 | 10.30 | 0.30 | 1.91E-03 |
| | VSIG4 | 8.86 | 10.84 | 0.29 | 9.08E-04 |
| | CD163 | 8.53 | 10.75 | 0.33 | 2.92E-04 |
| | CXCL14 | 6.99 | 9.48 | 0.44 | 1.11E-03 |

**Supplementary Table 4. The immune cells infiltration between GBM in and outside the VLSM-determined area.**

| Immune Cell | In the VLSM-determined area | | Outside the VLSM-determined area | | P Value |
|---|---|---|---|---|---|
| | Average | S.D. | Average | S.D. | |
| B cells naive | 0.01 | 0.04 | 0.01 | 0.02 | 7.50E-01 |
| B cells memory | 0.04 | 0.07 | 0.03 | 0.03 | 3.89E-01 |
| Plasma cells | 0.02 | 0.02 | 0.01 | 0.02 | 1.32E-01 |
| T cells CD8 | 0.04 | 0.04 | 0.01 | 0.02 | 1.21E-02 |
| T cells CD4 naive | 0.01 | 0.05 | 0.01 | 0.02 | 6.40E-01 |
| T cells CD4 memory resting | 0.05 | 0.07 | 0.06 | 0.05 | 4.51E-01 |
| T cells CD4 memory activated | 0.00 | 0.00 | 0.00 | 0.01 | 3.96E-01 |
| T cells follicular helper | 0.10 | 0.10 | 0.05 | 0.03 | 1.02E-02 |
| T cells regulatory (Tregs) | 0.01 | 0.03 | 0.01 | 0.01 | 1.78E-01 |
| T cells gamma delta | 0.02 | 0.03 | 0.02 | 0.04 | 5.15E-01 |
| NK cells resting | 0.00 | 0.01 | 0.01 | 0.02 | 1.20E-01 |
| NK cells activated | 0.03 | 0.04 | 0.02 | 0.03 | 3.77E-01 |
| Monocytes | 0.02 | 0.03 | 0.06 | 0.06 | 5.48E-02 |
| Macrophages M0 | 0.10 | 0.12 | 0.10 | 0.11 | 9.63E-01 |
| Macrophages M1 | 0.02 | 0.02 | 0.02 | 0.02 | 8.19E-01 |

| | | | | | |
|---|---|---|---|---|---|
| Macrophages M2 | 0.35 | 0.18 | 0.39 | 0.11 | 3.35E-01 |
| Dendritic cells resting | 0.01 | 0.03 | 0.00 | 0.01 | 2.87E-01 |
| Dendritic cells activated | 0.03 | 0.05 | 0.01 | 0.01 | 6.80E-02 |
| Mast cells resting | 0.02 | 0.02 | 0.01 | 0.02 | 2.87E-01 |
| Mast cells activated | 0.05 | 0.06 | 0.10 | 0.12 | 1.72E-01 |
| Eosinophils | 0.01 | 0.02 | 0.00 | 0.00 | 5.25E-02 |
| Neutrophils | 0.06 | 0.04 | 0.07 | 0.03 | 4.73E-01 |
