# Supplementary material for: A Voxel-Based Radiographic Analysis Reveals the Biological Character of Proneural-Mesenchymal Transition in Glioblastoma
Source: Front Oncol. 2021 Mar 17;11:595259. doi: 10.3389/fonc.2021.595259 (PMC8010193; doi:10.3389/fonc.2021.595259)
Supplement: Supplementary file 2 [file Image_1.PDF]

Supplementary Material

Supplementary Figures

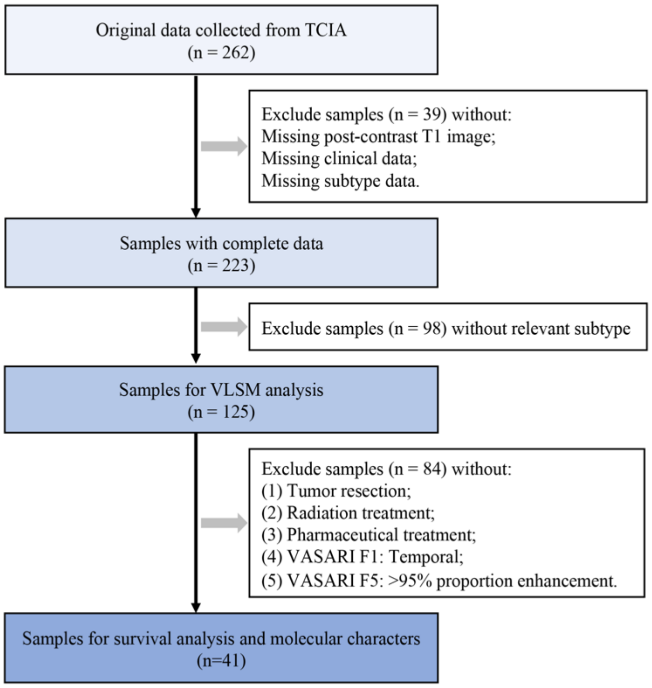

Supplementary Figure 1. Flowchart of patients included and excluded for analysis.
